# Role of the Insulin-Like Growth Factor Type 1 Receptor in the Pathogenesis of Diabetic Encephalopathy

**DOI:** 10.1155/2015/626019

**Published:** 2015-05-18

**Authors:** Duo Zhang, Shuang Jiang, Heng Meng

**Affiliations:** ^1^Department of Radiology, Affiliated Hospital of BeiHua University, JiLin 132011, China; ^2^College of Basic Medical Sciences, Changchun University of Chinese Medicine, Changchun, Jilin 130117, China

## Abstract

Defective cognitive function is common in patients with diabetes, suggesting that insulin normally exerts anabolic actions in neuron, namely, diabetic encephalopathy. However, because insulin can cross-activate the insulin-like growth factor type 1 receptor (IGF-1R), which also functions in most of tissues, such as muscle and bone, it has been difficult to establish the direct (IGF-1-independent) actions of insulin in the pathogenesis of diabetic encephalopathy. To overcome this problem, we examined insulin signaling and action in primary PC-12 cells engineered for conditional disruption of the IGF-1 receptor (ΔIGF-1R). The results showed that the lower glucose metabolism and high expression of IGF-1R occurred in the brain of the DE rat model. The results also showed the defect of IGF-1R could significantly improve the ability of glucose consumption and enhance sensitivity to insulin-induced IR and Akt phosphorylation in PC12 cells. And meanwhile, IGF-1R allele gene knockout (IGF-1R^neo^) mice treated with HFD/STZ had better cognitive abilities than those of wild mice. Those results indicate that insulin exerts direct anabolic actions in neuron-like cells by activation of its cognate receptor and prove that IGF-1R plays an important role in the pathogenesis of diabetic encephalopathy.

## 1. Introduction

The case reports are presented in the order of increasing severity of the neuropathological changes [1, 2]. The severe damage found on histological examination of the brains from the patients justifies the term encephalopathy [3–5]. One point of interest was whether cerebral changes were the cause or a sequela of the disease [6, 7]. Diabetes and its treatment are associated with functional and structural disturbances in the brain [8–10]. Many existing publications focused on changes in cerebral function and structure that develop more insidiously [10]. These changes are referred to as diabetic encephalopathy (DE), a term that encompasses functional impairment of cognition, cerebral signal conduction, neurotransmission and synaptic plasticity, and underlying structural pathology associated with diabetes [11–14].

Insulin-like growth factor-1 (IGF-1) that is a single-chain polypeptide is widely expressed in central nervous system [15–17]. Overexpression and genetic ablation of components of the IGF system in animal models can lead to developmental anomalies and functional disturbances [18–20]. IGF-1 acts primarily through its receptor, IGF-1 receptor (IGF-1R), which is widely distributed in the brain [21]. Binding of IGF-1 to IGF-1R may activate two major signaling pathways, PI3K/Akt and MAPK pathways [22–24]. The activated form of Akt, phosphorylated Akt (p-Akt), may inhibit several proapoptotic factors including glycogen synthase kinase-3 beta (GSK-3 beta), fork-head homolog in rhabdomyosarcoma (FKHR), Bcl-2-associated death protein, and caspase-9; each of them may influence neuronal survival after stroke [25]. Autophosphorylation increases the kinase activity of IR-type receptors by 3 orders of magnitude, enabling them to phosphorylate a number of substrate proteins and engender growth or metabolic responses [26, 27]. In addition to forming homodimers, IR and IGF-1R can form heterodimers with each other [28–30].

To examine the direct actions of insulin in diabetic encephalopathy (DE) and elucidate signaling pathways downstream of the IR, we used a model for conditional removal of the IGF-1R in vitro by adenoviral introduction of the Cre-recombinase to primary rat PC-12 cell derived from mice carrying floxed IGF-1R alleles. We show that PC-12 cells lacking the IGF-1R are two to three times more sensitive to insulin than are cells expressing both receptors. And in the model for downregulated IGF-1R in vivo, the knock-down (IGF-1R^neo^) mice treated with HFD/STZ have better cognitive abilities than those of wild mice. It is concluded that insulin exerts direct anabolic actions in neuron-like cells by activation of its cognate receptor; the solid data provided in the study proves that IGF-1R plays an important role of in the pathogenesis of DE.

## 2. Materials and Methods

### 2.1. Experimental Animals and Creation of Animal Model

Wistar rats (male, weighing 180–200 g) were supplied by the Laboratory Animal Center of BeiJing. All animal experiments were conducted according to the guidelines of the local animal use and care committees and executed according to the National Animal Law. The animals were divided into three groups: normal controls (CON, *n* = 25), diabetic encephalopathy (D, *n* = 25), and diabetic encephalopathy (DE, *n* = 25). The rats were fed with HFD for 4 weeks; STZ was prepared before each use at 20 mg/mL in 0.1 M pH 4.4 citrate buffer and was injected at 150 mg/kg, i.p., into rats which had been fasted for 12 h prior to receiving the injection. Four days later, nonfasting blood glucose in a tail-vein sample was determined by a glucose analyzer; a value >15 mM/L was accepted as a successfully created diabetic model.

The IGF receptor null (IGF-1R^−^) mice were not used in this study, because Epaud et al. reported that IGF-1R^−/−^ embryos displayed severe lung hypoplasia and markedly underdeveloped diaphragms, leading to lethal neonatal respiratory distress [30]. IGF-1R allele gene knockout (IGF-1R^neo^) mice described previously [6, 13, 21]. For studies in young adult mice, IGF-1R^neo^ mice were 9 to 12 weeks old. Mice lived under SPF conditions in individually ventilated filter-cages. All IGF-1R^neo^ mice were provided by Changchun ibiocc Co., Ltd. Preparation of HFD/STZ-induced diabetic mice refers to the publication [31].

### 2.2. Test of Abilities of Learning and Memory

Morris water maze tests were performed after training for 12 weeks. After the rats were familiar with the testing environment, normal training was performed from the second day. Orientation test: rats were trained twice per day, one time in the morning and one time in the afternoon. Each training lasted for 120 s and the gap time was 30 s. This training lasted for 4 days. Starting area was randomly selected and the number of times rats touch the platform in 120 s was recorded. The platform was removed, and the rats were put into water at the opposite side of the platform. The percent of residence time in the center area and number of times of passing the former platform in 120 s were recorded.

### 2.3. In Vivo PET Studies

PET studies were performed in the rats which were suffering from diabetes or diabetic encephalopathy (*n* = 20 per group). The PET protocol was the following report.

### 2.4. Biochemistry Markers Test

The brains of rats in each group after the test of abilities of learning and memory were collected on the ice and then the hippocampus was dissected. Tissues were crushed and centrifuged at the speed of 2000 r/min for 10 min. The supernate was collected and the activities of SOD, GSH-Px, CAT, and content of MDA in the rat hippocampal gyrus were detected. Coomassie brilliant blue staining was used to detect protein concentration.

### 2.5. HE Staining Test

Thirty *μ*m brain coronal sections were collected from every 200 *μ*m. The sections were deparaffinized, with two changes of xylene, 10 min each. They were rehydrated in 2 changes of absolute alcohol, for 5 min each, 95% alcohol for 2 min and 70% alcohol for 2 min, then washed briefly in distilled water, and stained in Harris hematoxylin solution for 8 min. They were washed in running tap water for 5 min and differentiated in 1% acid alcohol for 30 s. After this, they were washed in running tap water for 1 min and blued in 0.2% ammonia water or saturated lithium carbonate solution for 30 to 60 s. Again they were washed in running tap water for 5 min, rinsed in 95% alcohol, at 10 dips, and then counterstained in eosin-phloxine solution for 30 s. They were dehydrated in 95% alcohol, 2 changes of absolute alcohol, 5 min each. They were cleared in 2 changes of xylene, 5 min each, and mounted with xylene based mounting medium. The neurons in CA1 in hippocampus were observed using optical microscope.

### 2.6. IHC Staining Test

After dissecting tissues at 5 *μ*m and fixing them in 4% paraformaldehyde for 10 m, slides were incubated 2 to 3 times in xylene for 10 m each and then incubated twice in 100% ethanol for 2 m each. They were hydrated by placing in 95, 70, 50, and 30% ethanol for 2 min each. Slides were placed into buffer containing 5% normal goat serum for 10 min. Slides were incubated in a humidified chamber overnight with primary antibody (rabbit anti-rat Akt/PKB 1 : 500, rabbit anti-rat GLUT4 1 : 1000). They were washed in 5 m in buffer for 3 times and incubated with secondary antibody in a humidified chamber for 30 min. DAB and hematoxylin staining, 5 discontinue brain sections were selected and 5 fields were selected randomly. The numbers of Akt/PKB and GLUT4 positive cells in CA1 were counted.

### 2.7. Western Blot

Run 20 *μ*g protein per lane after heating at 100°C for 5 min. Run on an SDS-PAGE gel until the blue front is at the bottom of the gel. Transfer to a nitrocellulose membrane for 0.5 A-h. Block the membrane for 1 h in 5% nonfat dry milk in 1 × PBST, in a small Tupperware dish on a shaker. Incubate with primary antibody (rabbit anti-rat pAKT 1 : 500, rabbit anti-rat GLUT4 1 : 1000, and rabbit anti-rat *β*-actin 1 : 200) at 4°C overnight. Wash 3 times for 5 to 10 min in 50 mL 1 × PBS with 0.1% Tween 20 at RT. Incubate with goat anti-rabbit 1 : 200 for 1 h at RT in 1 × PBST, wash 3 × 10 min, and rinse with ddH_2_O. Detect protein with ECL kit (2 mL/membrane). In a separate tube, mix black and white ECL solutions in a 1 : 1 ratio. Then add aliquot solution onto membranes and wait for 1 min. Drain the ECL, wrap in plastic, and expose to film. The value of protein would be compared with *β*-actin, and the relative potency ratio would stand for the expression of protein.

### 2.8. Statistical Analysis

Data were expressed as mean ± standard deviation (M ± SD). Group differences in the swimming time in the Morris water maze test and the number of errors in the passageway water maze test were analyzed by SPSS 11.0 using Windows software to conduct two-way analysis of variance (ANOVA, equal variances assumed by S-N-K) on repeated measurements. Other data were analyzed by SPSS 11.0 using Windows software to conduct one-way ANOVA (equal variances assumed by S-N-K). A post hoc test was used to obtain the *P* values. *P* < 0.05 was considered significant.

## 3. Results

### 3.1. Comparation of Study and Memory Ability

**Figure 1:**
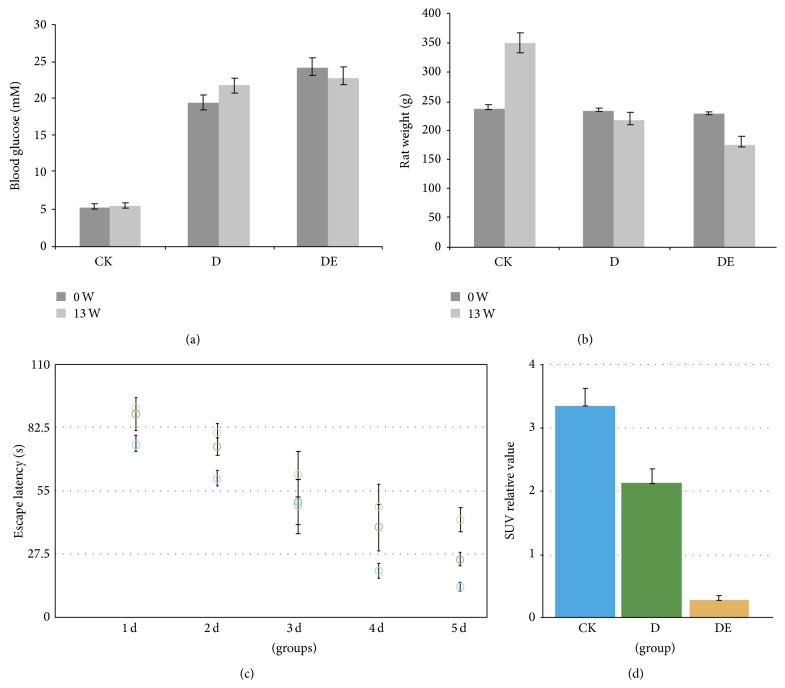
Blood glucose (a) and body weight (b) of mice in 3 groups. The ability analysis of learning and memory of mice in 3 groups (c). Evaluation of brain glycometabolism in DE animals by PET/CT (d).

The rats of D group and D + DE were with polydipsia, polyphagia, polyuria and weight loss, yellowish color, and poor spirit of the late, slow-moving symptoms. As shown in Figure 1, at the beginning of generating animal model, the values of blood glucose in D group and DE groups were much higher than Normal Control group in the 13th week (*P* < 0.01); the body weight of mice in 3 groups showed the same situation (*P* < 0.01).

During the training period, the escape latency in all rats decreased significantly as training days increased (F day = 1324.66, *P* < 0.01). To use the Morris water maze test, the rats in DE group had more swimming time than that in D group (*P* < 0.05) and made significantly more errors compared with that of Normal Control group (*P* < 0.05). The rats showed reversed behavioral alternation with levels returning close to that of rats in the control group (Figure 1(c)).

### 3.2. The Lower Glucose Metabolism in the Brain of the DE Rat Model

To investigate glucose metabolism in the DE rat model, the changes of [^18^F]-FDG-PET images were recorded. A significant positive correlation was found between D group and DE group and the [^18^F]-FDG uptake in the cortex and the hippocampus. Evaluation of glucose metabolism in animals revealed a decrease of cortical and hippocampal glucose uptake in the DE group compared with Normal Control group. In D group, more glucose was consumed, as compared with DE group. Based on the PET/CT data, low levels of glucose metabolism may be an important factor in the process of encephalopathy induced by diabetes (Figure 1(d)).

### 3.3. Abnormally High Expression of IGF-1R Occurs in the Brain Tissue of Rats Suffering from Diabetic Encephalopathy

**Figure 2:**
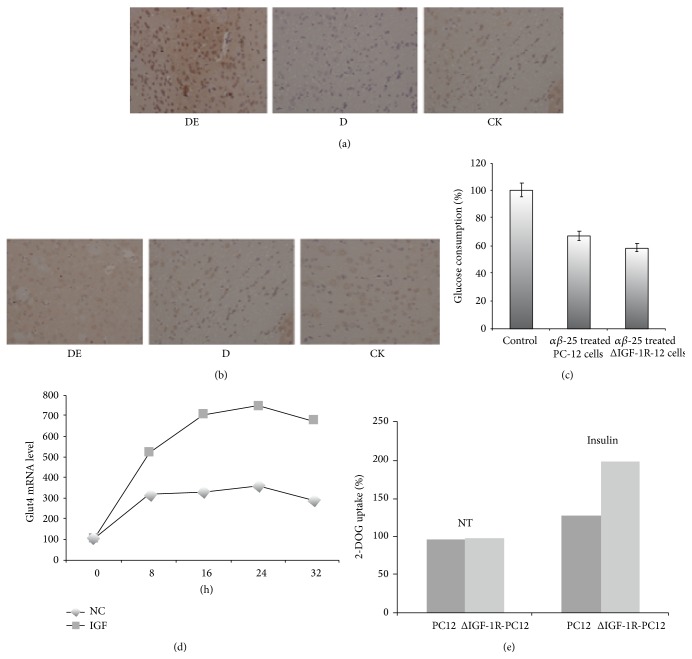
Expression of enhanced IGF-1R (a) and IR (b) in the hippocampal gyrus of DE rats. Enhanced insulin-induced glucose transport in ΔIGF-1R PC-12 cells (c, d, and e).

IGF-1R was measured by immunohistochemistry assay in DE group and D group. As is shown in Figures 2(a) and 2(b), compared with Normal Control group, it was found a sharp increase of IGF-1R in DE group, but there was a decrease in the diabetic group. On the other hand, no difference of IGF-1R expression was seen among the Normal Control group, DE group and D group in Figures 2(a) and 2(b) using the same method. Abnormally high expression of IGF-1R has been found in the studies for the organizations rarely. This phenomenon was ever confusing to us, so we decided to construct the transformed PC12 cell that expressed IGF-1R lower than normal cells. Speculation confirmed earlier that low IGF-1R expression is good for the glucose metabolism of neurons cell.

### 3.4. The Defect of IGF-1R Significantly Improved the Ability of Glucose Consumption in PC12 Cells

It is known that A*β*25–35 peptide has toxicity to affect the ability of glucose consumption of PC12 cell. At the resting state (no insulin), glucose consumption of PC12 ΔIGF-1R cell and PC12 cell has no significant increase or decrease on the four time points (*P* > 0.05).

In Figure 4(a), compared with Normal Control group, PC12 cells and ΔIGF-1R-PC12 pretreated with A*β*25–35, the amount of glucose that was consumed by these two cells was decreased; in particular, PC12 cells treated with A*β*25–35 have shown the weakest glucose consumption capacity. Here it is important that ΔIGF-1R-PC12 cells consumed the glucose more than that of PC12 cell treated with A*β*25–35. The data suggest that the defect of IGF-1R significantly improved the ability of glucose consumption in PC12 cells but still did not completely reverse the damage caused by A*β*25–35.

At the resting state (no insulin) both of cell groups have no significant increase or decrease (*P* > 0.05) of glucose consumption on the four time points. As is shown in Figure 4(c), compared with PC12 cells, in insulin-pretreated PC12 cells and ΔIGF-1R-PC12 cells incubated by A*β*25–35, glucose uptake capacity was significantly improved by the defect of IGF-1R (*P* < 0.05), suggesting the defect of IGF-1R promotes mRNA level of Glut4 and the uptaking of glucose significantly in the insulin-stimulated PC12 cells (Figures 2(c), 2(d), and 2(e)).

### 3.5. The knock-Down (IGF-1R^neo^) Mice Treated with HFD/STZ Have Better Cognitive Abilities Than Those of Wild Mice

Due to operational difficulties in molecular biology, we have not been able to use the rat model with lower expression of IGF-1R. The knock-down (IGF-1R^neo^) mice were made applying some similar methods which were reported [30].

Considering respiratory failure and exhibiting a more severe growth deficiency in lung, null IGF-1R^−^ Mice have not been used as a model in the study. Using (PE) for 10 (for each treatment group) Histology of the overall digital analysis in hippocampus, compared to wild-type mice models, the knock-down (IGF-1R^neo^) mice were significantly reduced in the control group that was approximately 33%. Due to insufficient accuracy of our PET-CT for animal, it cannot be used to detect levels of glucose metabolism in mice of the head; it is so regret in the study. Fortunately, the results of body weight, blood glucose, and cognitive abilities indicate the weight of knock-down IGF-1R^neo^ mice fed with high-fat high-sugar is lower than that of wild-type mice; this may be a positive tip that the IGF-1R^neo^ mice have a greater ability of glucose metabolism. More important is the knock-down (IGF-1R^neo^) mice blood glucose levels were significantly lower than the wild type, with statistical significance (*P* < 0.05), but still higher than the normal diet of knock-down (IGF-1R^neo^) mice (*P* < 0.01). As is shown in Tables 1 and 2, Morris water maze tests revealed the most important experimental result; only 2 knock-down (IGF-1R^neo^) mice showed slight cognitive barrier. However, 7 mice in wild-type mice fed with high-sugar high-fat treatment group had three in a serious cognitive disorder. Until testing the latter (25w), the living state of all wild mice was not suitable for water Morris maze test, while no knock-down (IGF-1R^neo^) mice still have the athletic ability and cognitive ability.

In summary, the low expression of IGF-1R could be help to inhibit diabetic encephalopathy to some extent.

### 3.6. Loss of the IGF-1R Enhances Sensitivity to Insulin-Induced IR Phosphorylation

**Figure 3:**
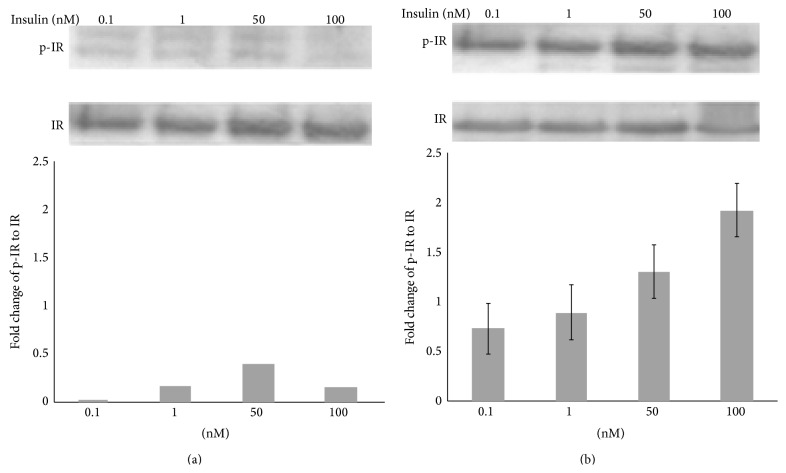
Selective insulin-induced phosphorylation of IRS-1 in ΔIGF-1R PC-12 cells (a, b).

To investigate the effects of IGF-1R disruption on immediate IR signaling, we examined IR autophosphorylation in the cells. The ΔIGF-1R and control cells were serum-starved and treated with insulin, lysed, and immunoprecipitated with the anti-IR-*β* antibody. Western blot analysis with an anti-Tyr (P) antibody showed that insulin stimulated tyrosine phosphorylation of the insulin receptor in both ΔIGF-1R and control cells (Figures 3(a) and 3(b)). However, in the ΔIGF-1R cells, IR was more responsive to insulin, leading to IR autophosphorylation at 100-fold lower insulin concentrations (0.1 nM) when compared with equivalent activation in control cells at the concentration of 10 nM.

### 3.7. Loss of the IGF-1R Enhances Sensitivity to Insulin-Induced Akt Phosphorylation

**Figure 4:**
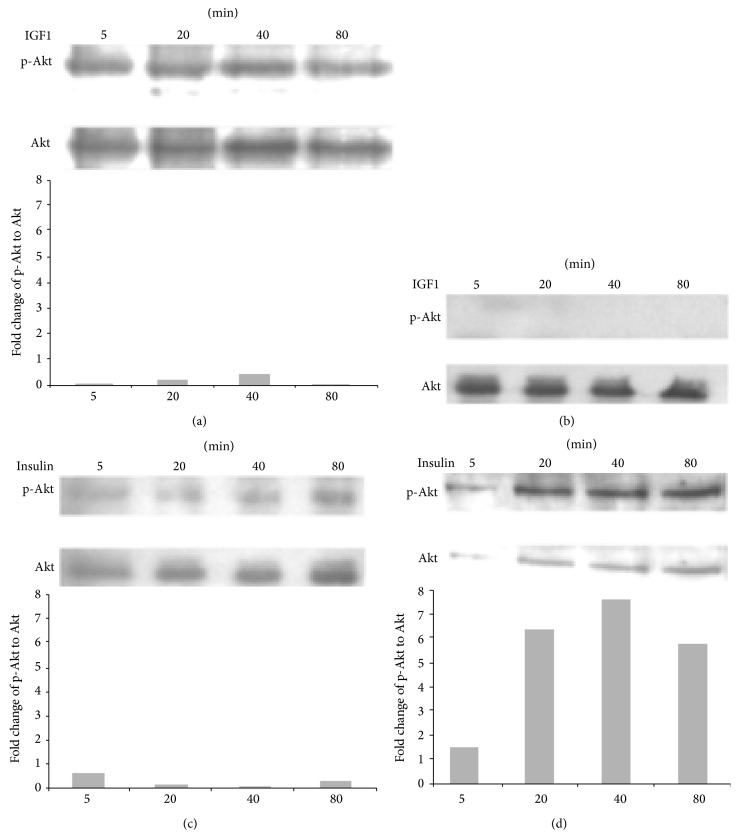
Loss of the IGF-1R enhances sensitivity to insulin-induced (a) and IGF1 (b) Akt phosphorylation.

Ligand binding to IR and IGF-1R activates Akt signaling pathways. As shown in Figure 4, IGF-1 acutely stimulated Akt (Figures 4(a) and 4(b), left panels) in control cells, whereas these effects were nearly abolished in ΔIGF-1R cells. Interestingly, insulin treatment resulted in significantly greater induction of Akt phosphorylation in PC12-ΔIGF-1R cells compared with control cells. The enhanced insulin sensitivity in ΔIGF-1R cells is opposite to that observed for growth hormone signaling where removal of the IGF-1R diminishes growth hormone induction of JAK/STAT phosphorylation.

## 4. Discussion

Diabetic encephalopathy is an unknown diabetes complication, characterized by electrophysiological, structural, neurochemical, and degenerative neuronal changes that lead to cognitive functioning limitations. Hence it is named as “type 3 diabetes”; the content of this title represents the most relevant risk factor for increased incidence of dementia, cognitive dysfunction, and consequently Alzheimer's disease.

As is known widely, insulin is an important anabolic hormone identified, since almost all of cell types are sensitive to this peptide [32]. More and More evidence proved that the hormone is widely located in the brain [32]. It plays a critical and central role in numerous actions in the brain, like neurotrophic, neuromodulatory, and neuroendocrine [33–35]. Additionally, insulin runs in the CNS through binding to the receptors on cell membrane—insulin receptor (IR) and insulin growth factor-1 receptor (IGF-1R); they are so abundant throughout the whole brain, such as hypothalamus, hippocampus [36, 37]. Once bound to the receptors, insulin triggers signaling cascades that include PI-3K and Akt pathways, which are the most relevant factors involved in learning and memory processes [38].

Insulin-like growth factor-1 receptor (IGF-1R) locates on the cell types in many human tissues [39]. Two peptide hormones called IGF-1 and IGF-2 both can activate it effectively. Their actions are mostly like insulin. Both of them have anabolic effects in adults—meaning that it can induce hypertrophy of brain and other target tissues. IGF-1R and other tyrosine kinase growth factor receptors signal through multiple pathways. In Figure 3, the results of Western blot showed that IR on ΔIGF-1R cells was more susceptible to insulin, so the pathways between IR and IGF-1R may cross and overlap; if one of them defects, the other will operate alternately. A key pathway is regulated by phosphatidylinositol-3 kinase (PI3K) and its downstream partner, the mammalian target of rapamycin. IGF-1 prosurvival action is mainly activated by the PI3K/Akt pathway [40, 41]. PI3K inhibitors or expression of an inactive Akt mutant can suppress the neuroprotective effects of IGF-1, supporting the hypothesis that the survival signal is mediated predominantly through this pathway. Furthermore, some inflammatory factors, such as tumor necrosis factor- (TNF-) alpha can also indirectly trigger the death of neurons by inhibiting essential components of the IGF-1 survival response, such as PI3K, further demonstrating the key role of the IGF-1/PI3K-Akt pathway in neuroprotection.

Activation of PI3K stimulates the phosphorylation of the survival kinase, Akt. Activated Akt can phosphorylate multiple downstream proteins related to cell survival. This is consistent with a recent study, which demonstrated that no regional or aging difference was observed in total Akt level, but activated Akt was significantly reduced in hippocampal CA1 region [42–45]. As shown in Figure 4, IGF-1 in ΔIGF-1R cell had no effects on ΔIGF-1R cells. On the other hand, more insulin-sensitivity was identified in ΔIGF-1R cells than the cells in control. These results suggest that the decrease of p-Akt signaling is related to the vulnerability of CA1 neurons to stressor such as ischemia.

## 5. Conclusion

It is concluded that insulin exerts direct anabolic actions in neuron-like cells by activation of its cognate receptor and proves that IGF-1R plays an important role of in the pathogenesis of diabetic encephalopathy.

## Figures and Tables

**Table 1:** Grade analysis of the pass-through time of mice in the water maze Automatic Control System test.

*T*_*p*_ − *T*/*T*_*p*_	Wild-mice HFD (*n* = 18)	IGF-1R^neo^ mice HFD (*n* = 18)
0%~20%	11	16
20%~30%	3	2
30%~40%	3	0
>40%	1	0

*T*_*p*_ = the pass-through time; *T* = the middle time of negative control.

**Table 2:** The times of error in MS-2 water maze Automatic Control System test.

Groups	Times of error			
	Day 1	Day 2	Day 3	Day 4
NS control group	9.12 ± 8.01	7.50 ± 5.56	5.43 ± 4.42	3.65 ± 2.21
Wild-mice HFD	15.08 ± 5.13	15.71 ± 10.15	12.13 ± 5.70	7.30 ± 2.11
IGF-1R^neo^ mice HFD	11.12 ± 7.01	8.50 ± 4.11	7.24 ± 4.57^*^	5.88 ± 2.46^*^

^∗^ *P* < 0.05; *n* = 18 mice per group.
